# Spexin Acts as Novel Regulator for Bile Acid Synthesis

**DOI:** 10.3389/fphys.2018.00378

**Published:** 2018-04-10

**Authors:** Cheng-yuan Lin, Ling Zhao, Tao Huang, Lin Lu, Mahjabin Khan, Jie Liu, Linda L. D. Zhong, Zong-wei Cai, Bao-min Fan, Anderson O. L. Wong, Zhao-xiang Bian

**Affiliations:** ^1^Lab of Brain and Gut Research, School of Chinese Medicine, Hong Kong Baptist University, Kowloon Tong, Hong Kong; ^2^YMU-HKBU Joint Laboratory of Traditional Natural Medicine, Yunnan Minzu University, Kunming, China; ^3^Department of Chemistry, Hong Kong Baptist University, Kowloon Tong, Hong Kong; ^4^School of Biological Sciences, University of Hong Kong, Hong Kong, China

**Keywords:** bile acid, CYP7A1, spexin, ultraperformance liquid chromatography mass spectrometry, metabolism

## Abstract

Spexin is a novel hormone involved in obesity and diabetes while its biofunctional significance in lipid metabolism is still to be comprehended. Global metabolomic analysis in the present study revealed multiple metabolic pathways altered by spexin intraperitoneal (*i.p*.) injection in rat serum, which are highlighted by the changes in several bile acid metabolites. In rats, spexin (300 μg/kg) could dramatically reduce hepatic and circulating total bile acids (TBA) level compared with the controls. Correspondingly, treatment with spexin by *i.p*. injection for 28 days led to significant decrease in serum TBA and gallbladder weight in C57BL/6J mice. In enterohepatic circulation system, spexin effectively reduced TBA levels in mouse liver and gallbladder but not the intestine. Hepatic cholesterol 7α-hydroxylase 1 (CYP7A1) expression, unsurprisingly, was suppressed by spexin injection. Both GALR2 and GALR3 antagonists reversed the inhibitory effects of spexin on concentrations of serum TBA and 7 α-hydroxy-4-cholesten-3-one (C4), and hepatic CYP7A1 expression. Finally, negative correlations were observed between serum spexin and total cholesterol (TC), total bile acid (TBA), tauro-chenodeoxycholate (TCDCA), as well as glycochenodeoxycholate (GCDCA) in 91 healthy volunteers. These findings illuminate the intrinsic importance of spexin in the regulation of bile acid synthesis and metabolism.

## Introduction

Spexin, an endogenous peptide discovered using bioinformatics tools (Mirabeau et al., 2007; Sonmez et al., 2009), has been found to activate both galanin receptor 2 (GALR2) and galanin receptor 3 (GALR3) (Kim et al., 2014). Extensive distribution of spexin, GALR2 and GALR3 in various tissues from central nervous system to peripheral tissues (Waters and Krause, 2000; Porzionato et al., 2010; Wong et al., 2013) indicates that spexin might have multiple biological functions. Shortly after being discovered, spexin was postulated to regulate gut motility for its ability to induce rat stomach smooth muscle contractions and mouse intestinal motility (Mirabeau et al., 2007; Lin et al., 2015). In fish model, spexin was found to be a suppressor for food intake (Wong et al., 2013; Li et al., 2016; Wu et al., 2016; Ma et al., 2017). Recent studies in rodents unveil the physiological role of spexin in obesity and diabetes (Walewski et al., 2014; Gu et al., 2015), which is supported by the clinical observations that circulating level of spexin is negatively correlated with blood glucose in type 2 diabetes mellitus (T2DM) patients (Gu et al., 2015) and body weight in obese children (Kumar et al., 2016). In mice, spexin treatment effectively suppressed hepatic lipids and long-chain fatty acid (LCFA) uptake, contributing to loss in body weight (Ge et al., 2016). These findings imply that spexin may be an intrinsic regulator of glucose and lipid metabolism in obesity and T2DM condition.

Recent studies involving bile acid signaling regulation of glucose and lipid metabolism suggest that impaired bile acid metabolism may contribute to the pathogenesis of obesity and T2DM (Li and Chiang, 2012; Chavez-Talavera et al., 2017). Bile acids are major products of cholesterol catabolism, and play important roles in liver and intestinal diseases (Hofmann, 2007). In the liver, bile acids are synthesized by the regulation of over 10 enzymes followed by conjugation to glycine or taurine (Chiang, 2009; Russell, 2009). The major bile acid pathway is initiated by cholesterol 7α-hydroxylase (CYP7A1), a cytochrome P450 enzyme located in the endoplasmic reticulum of the liver (Chiang, 2009). The produced conjugated and unconjugated bile acids are released into the intestine through the bile duct and reabsorbed in the ileum to the portal blood for circulation back to the liver, whereas small portion of bile acids (about 5%) are propelled into the colon along with intestinal contents and excreted with the feces (Thomas et al., 2008; Chiang, 2009; Boyer, 2013). Farnesoid X receptor (FXR) and G protein-coupled receptor TGR5 are the two major receptors widely distributed in the liver and gastrointestinal tract for feedback regulation of bile acid synthesis and other metabolic pathways and physiological functions (Thomas et al., 2008; Schaap et al., 2014). Li et al. reported that Type II diabetic ob/ob mouse exhibited increased basal expression of CYP7A1 and bile acid pool (Li et al., 2012). Interestingly, CYP7A1-transgenic mice were found to be resistant to high fat diet-induced insulin resistance and obesity (Li et al., 2010). This is consistent with another finding that reduction of bile acid pool size may facilitate the development of diabetes and obesity which could be reversed by increasing the bile acid pool size (Watanabe et al., 2011). It is probable that simultaneous activity of other endogenous factors may contribute to these complex observations which vary with different research methods. Further extensive studies on the intrinsic regulators of bile acid synthesis are required to warrant the elucidation of pathogenesis of diabetes and shed light on potential therapeutic strategies for the disease.

In this study, we conducted untargeted metabolomic study which unveiled several categories of significant features in serum metabolome of spexin-injected vs. saline-injected rats. It is worth mentioning that the metabolite set of bile acids was enriched in the serum of spexin-injected rats, implying the important spexin biological actions on bile acid metabolism. In this case, the effect of spexin on bile acid profile in the enterohepatic system was investigated using ultrahigh performance liquid chromatography-triple-quadrupole mass spectrometry (UPLC-TQ/MS) analysis. *In vivo* studies demonstrated the inhibitory action of spexin on bile acid synthesis via CYP7A1 repression, which could effectively be reversed by GALR2/3 blockade. The results disclose novel physiological functions of spexin on bile acid metabolism and provide new insights for further studies on the lipid signaling pathogenesis of metabolic diseases.

## Materials and methods

### Chemical and reagents

A series of bile acid standards, containing cholate (CA), chenodeoxycholate (CDCA), ursodeoxycholate (UDCA), deoxycholate (DCA), lithocholate (LCA), glycocholate (GCA), glycochenodeoxycholic acid (GCDCA), glycodeoxycholate (GDCA), taurocholate (TCA), taurodeoxycholate (TDCA), tauroursodeoxycholate (TUDCA), taurohyodexoycholate (THDCA), taurochenodeoxycholate (TCDCA), taurolithocholate (TLCA) were purchased from Sigma-Aldrich (St. Louis, MO, USA). Tauro-β-muricholate (TβMCA) was purchased from Santa Cruz Biotechnology (Santa Cruz, CA, USA). As internal standard (IS), deoxycholic acid-2,2,4,4-d4 (DCA-d4) were obtained from CDN isotopes (Pointe-Claire, Quebec, Canada). All organic reagents for mass spectrometric analysis were HPLC grade purchased from Sigma-Aldrich (St. Louis, MO, USA). And spexin used for drug treatment was purchased from Phoenix Pharmaceuticals (Belmont, CA, USA). M871 was purchased from R&D Systems (Minneapolis, MN, USA). SNAP37889 was purchased from Key Organics Ltd (Camelford, Cornwall, UK).

### Animals and spexin *i.p*. injection

Male Sprague-Dawley rats weighing 200–250 g and male C57BL/J mice weighing 20-25 g were obtained from the Laboratory Animal Services Center, The Chinese University of Hong Kong, Hong Kong. The animals were fed with a standard rodent diet *ad libitum* with free access to water and were housed in rooms maintained at 22 ± 1°C with a 12 h light/dark cycle (lights on 6:00-18:00). The Animal Ethics Committees of Hong Kong Baptist University, approved all experimental protocols, in accordance with “Institutional Guidelines and Animal Ordinance” from Department of Health, Hong Kong Special Administrative Region.

#### Spexin *i.p*. injection in rats

Rats were injected *i.p*. with spexin prepared in saline. The dose of spexin were 300 μg/kg according to previous studies (Lin et al., 2015). One hour later, the rats were anesthetized with CO_2_. The rats injected *i.p*. with saline were served as control group. Blood, liver, ileum, colon, luminal contents, and feces were harvested. All the samples were snap frozen in liquid nitrogen and stored in −80°C until the further analysis. For untargeted metabolic profile analysis, six rats were used in each group. And for quantification of bile acid metabolites, eight rats were used in each group.

#### Long-term treatment of spexin in mice

All mice were acclimated to the facility for 1–2 week before the experiments and randomly divided into three groups (*n* = 10 per group): (1) vehicle (normal fed, receiving daily *i.p*. saline), (2) spexin-L (receiving daily *i.p*. 12.5 μg/kg spexin), (3) spexin-H (receiving daily i.p. 25 μg/kg spexin). The doses were set according to the previous studies (Ge et al., 2016). The mice were treated with spexin or saline in the morning of each day. Body weight was monitored every 4 days. The treatment of spexin were continuously conducted until the body weight gain showed consistently significant difference between the control group and spexin-treated groups. At the end of the experiment (Day 28), blood, liver, gallbladder, and ileum contents were harvested. Serum and all tissue samples were snap frozen in liquid nitrogen and stored in −80°C until further analysis.

#### Treatment of spexin antagonists

All mice were acclimated to the facility for 1–2 week before the experiments and randomly divided into six groups (*n* = 10 per group): (1) vehicle (*i.p*. with saline), (2) spexin (*i.p*. with spexin 300 μg/kg), (3) M871 (*i.p*. with M871 1000 μg/kg), (4) spexin +M871 (*i.p*. with M871 15 min before spexin treatment), (5) SNAP37889 (*i.p*. with SNAP37889 1 mg/kg), (6) spexin+SNAP37889 (*i.p*. with SNAP37889 15 min before spexin treatment). One hour after drug treatment, serum and liver samples from each mouse were collected, snap frozen in liquid nitrogen and stored in −80°C for further analysis.

### Extraction of bile acid metabolites from diverse specimens

#### Serum

The procedure of bile acid extraction in different specimens was performed as described in previous study (Xie et al., 2013). Briefly, for serum bile acid extraction, four-fold mixture of methanol (containing 50 ng/mL of DCA-d4 as the internal standard) was added into 50 μL of serum samples for protein precipitation and bile acid extraction. After vortex (standing for 10 min) and high-speed centrifugation (13,000 rpm for 15 min) at low temperature, the supernatant was transferred into a new tube for further targeted profile analysis.

#### Tissues

Tissue samples (liver and intestine), precisely weighed at 50 mg, were homogenized in a 300 μL volume of mixed solvent with water, methanol, and chloroform (1:2.5:1, v/v/v). Then, the mixture was vortexed and centrifuged under the same conditions as the procedure in serum extraction prior to transferring 150 μL of the supernatant to a new tube. The depositing tissues were homogenized again with 300 μL volume of methanol, and another 150 μL of the supernatant was combined with the previous one. The supernatants with 50 ng/mL of DCA-d4 were dried under nitrogen and re-dissolved with methanol for further analysis.

#### Luminal contents

An aliquot of 500 μL of deionized water was used to extract bile acids in luminal contents or feces (100 mg). The mixtures were adequately vortexed (for 5 min) and centrifuged (13,500 rpm for 15 min) at 4°C. After transferring 300 μL supernatants into new tubes, the pellets were mixed with 500 μL of methanol. The same volume of supernatants from twice extractions (containing 50 ng/mL of DCA-d4 as the internal standard) were mixed, then vortexed and centrifuged according to previous protocol. The final supernatants were then extracted for further analysis.

### Untargeted metabolic profile

The untargeted metabolic analysis was performed as described previously (Zhao et al., 2017). The four volumes of cold methanol containing 5 μg/mL of p-chlorophenylalanine solution as IS were added into 50 μL of serum for metabolites extraction and protein precipitation. Samples were vortexed and centrifuged at 13,000 rpm for 10 min. The 200 μL of supernatant was dried and redissolved in the same volume of solvent consisting of water and acetonitrile (98:2, v/v).

The 2 μL of resulting supernatant was injected into a liquid chromatography system (UPLC, Agilent 1290 Infinity, USA) and separated by gradient elution with 0.35 mL/min of flow rate using ACQUITY UPLC BEH C18 column (1.7 μm, 2.1 × 50 mm, Waters Corporation, Milford, MA). The gradient program consisted of phase A (0.1% formic acid in water) and phase B (0.1% formic acid in acetonitrile), which started from 2 to 5% B in 1 min, then raised to 100% B in next 11 min and maintained at 100% B for 3 min, finally turned back to 2% B in 2 min. A quadruple time-of-flight mass spectrometer (Q-TOF/MS, Agilent 6543, USA) coupled with electrospray ionization (ESI) was performed for acquisition of metabolic fragments in both positive and negative ionic modes. The instrument operated in full scan mode from 100 to 1,000 m/z and the capillary voltage was set at 3,000 V.

### UPLC/MS-based quantification of bile acid metabolites

#### Stock solution and calibration curve preparation

Fourteen bile acid chemical standards were separately dissolved in methanol as stock solution with a concentration of 5 mg/mL. A mixed stock solution was obtained after mixing individual standard stock solution. Diluting stock solutions in methanol, the working solution were prepared at a series concentration of 0.020, 0.102, 0.512, 2.56, 12.8, 64, 320, 1,600, 8,000, and 40,000 ng/mL for bile acid metabolites, while at a series concentration of 0.064, 0.32, 1.6, 8, 40, 200, 1,000, and 5,000 ng/mL for serum C4. The standard curves and regression coefficients were gained based on IS adjustment. The signals of each bile acid metabolites were found in individual measured ranges.

#### UPLC/TQ-MS condition and bile acid analysis

All bile acid extractions from specimens were analyzed with referral to a previous method, with minor modification in ion acquisition (Xie et al., 2015). Briefly, an ultra-high-performance liquid chromatography (Agilent UHPLC 1290, USA) coupled with a triple-quadrupole mass spectrometer (Agilent QQQ-MS 6438, USA) was applied for bile acid analysis. Though a single 26-min acquisition with positive/negative ion switching, bile acid metabolites (under ESI-) and C4 (under ESI+) were simultaneously quantified in multiple reaction monitoring (MRM) mode. Sample injection and flow rate were set at 2 μL and 0.35 mL/min for each sample, respectively. Bile acid metabolites were separated using a ACQUITY BEH C18 column (1.7 μm, 100 × 2.1 mm) with a linear gradient of 0.1% formic acid (FA) in water (A) and 0.1% FA in acetonitrile (B). The gradient program was: 25–40% B for the first 6 min, 40–70% B for 14 min, 70–100% B for 0.1 min, held at 100% B for 2.9 min, then re-equilibration at 25% B for 0.1 min, and held at 25% B for 2.9 min. The column temperature was maintained at 45°C. The capillary voltage of mass spectrometer was 3.5 and 4 kV in positive and negative modes. The investigated metabolites along with their specific MRM transitions and MS/MS parameters were shown in Table S1. The acquisition data was analyzed using Agilent MassHunter Workstation Software for peak integration, calibration equations, and quantification of individual BAs.

### Quantitative real-time PCR analysis

Mice liver and ileum tissues (50 mg) were homogenized with TissueLyzer (Qiagen, Hilden, Germany), and the total RNA was isolated with TRIZOL (Life technologies, Invitrogen, Carlsbad, CA, USA). The cDNA synthesis was performed with the SuperScript® First-Strand synthesis system for RT-PCR (Invitrogen, Carlsbad, CA, USA) according to the manufacturer's instruction. Power SYBR Green Master Mix (Applied Biosystems, Foster city, CA, USA) was used for the quantitative real-time PCR on the ViiA™ 7 Real-Time PCR System (Applied Biosystems, Foster city, CA, USA). Gene-specific primers synthesized by Thermo Fisher Scientific (Invitrogen, Carlsbad, CA, USA) were used and are listed in Table 1. The targeted gene expressions were normalized to corresponding β-actin level and further analyzed with ΔΔC_T_ analysis method.

**Table 1:** Primer sets for quantitative RT-PCR analysis of mouse genes.

**Gene**	**Abbreviation**	**Sequence**
Cytochrome P450 7A1	CYP7A1	AGCAACTAAACAACCTGCCAGTACTA
		GTCCGGATATTCAAGGATGCA
Cytochrome P450 7b1	CYP7b1	TAGCCCTCTTTCCTCCACTCATA
		GAACCGATCGAACCTAAATTCCT
Cytochrome P450 27A1	CYP27A1	GCCTCACCTATGGGATCTTCA
		TCAAAGCCTGACGCAGATG
Cytochrome P450 8b1	CYP8b1	GGCTGGCTTCCTGAGCTTATT
		ACTTCCTGAACAGCTCATCGG
Bile acid CoA: amino acid *N*-acyltransferase	BAAT	GGAAACCTGTTAGTTCTCAGGC
		GTGGACCCCCATATAGTCTCC
Hepatocyte nuclear factor 4 alpha	HNF4α	AAATGTGCAGGTGTTGACCA
		CACGCTCCTCCTGAAGAATC
Organic solute transporter alpha	OSTα	TGTTCCAGGTGCTTGTCATCC
		CCACTGTTAGCCAAGATGGAGAA
Organic solute transporter beta	OSTβ	GATGCGGCTCCTTGGAATTA
		GGAGGAACATGCTTGTCATGAC
Na^+^-taurocholate cotransporting polypeptide	NCTP	ATGACCACCTGCTCCAGCTT GCCTTTGTAGGGCACCTTGT
Small heterodimer partner	SHP	CGATCCTCTTCAACCCAGATG
		AGGGCTCCAAGACTTCACACA
Farnesoid X Receptor	FXR	TGTGAGGGCTGCAAAGGTT
		ACATCCCCATCTTGGAC
Fibroblast growth factor receptor 4	FGFR4	GCCTCCGACAAGGATTTGGCA
		GAGTGCAGACACCCAGCAGGT
Apical sodium dependent bile acid transporter	ASBT	ACCACTTGCTCCACACTGCTTCGTTCCTGAGTCAACCCACAT
Fibroblast growth factor	FGF15	ACGTCCTTGATGGCAATCG
		GAGGACCAAAACGAACGAAATT
Beta actin	β-actin	ACCTGACAGACTACCTCATGAAGA
		TCATGGATGCCACAGGATTCCATA

### Measurement of serum total bile acid (TBA), alkaline phosphatase (ALP), and total cholesterol (TC)

Serum TC level was determined using reagent kits purchased from Abcam (Cat No: ab65390; Cambridge, UK). TBA level in human serum was measured using commercial Total Bile Acid Assay Kit (Cell Biolabs, San Diego, CA, USA). Serum ALP level was examined using Alkaline Phosphatase Assay Kit (Abcam, Cambridge, UK).

### Confocal imaging of mouse liver samples

Mouse liver slices were fixed in 4% PFA in PBS overnight at 4 °C and embedded in paraffin. The slides were prepared and processed to immunofluorescence staining as previously described (Xiao et al., 2017). Images were acquired on a Leica TCS SP8 laser confocal microscope. Primary and secondary antibodies used: rabbit anti-spexin (Bachem, T-4858, 1:500), rabbit anti-GALR2 (Aviva Systems Biology, OABF00517, 1:100), rabbit anti-GALR3 (Aviva Systems Biology, OAAF04899, 1:100), AlexaFluor 594-conjugated goat anti-rabbit IgG (Thermofisher Scientific, R37117, 1:500). Fluoromount-G™, with DAPI (Invitrogen, 00-4959-52) was used as fluorescent nuclear stain and mounting slides.

### Healthy volunteers and serum samples

Ninety-one healthy adults were recruited in clinical division at the School of Chinese Medicine, Hong Kong Baptist University, Hong Kong, China. The recruiting criteria for healthy population is shown as follows:

*Inclusion criteria:* (1) age of 18–65 years (inclusive); (2) no medical history of metabolic disorders, cardiovascular diseases, neurodegenerative diseases, and gastrointestinal diseases; (3) Normal hepatic, renal, and bowel functions within 3 years; (4) No drug taken history for chronic diseases, metabolic diseases, cardiovascular and cerebrovascular diseases, psychiatric illness, disease of immune system, and other serious diseases within 1 year, (5) written informed consent.
*Exclusion criteria:* (1) pregnancy or breast-feeding; (2) surgical histories of gallbladder removal, GI tract, and cerebral cranium; (3) Use of medications known to influence gastrointestinal transit, blood pressure, and fat.

This study was approved by the Hong Kong Baptist University Ethics Committee on the Use of Human Subjects for Teaching and Research (Approval no. HASC/16-17/0027). All participants signed an informed consent form for such project. Several biochemical indexes were testified at the Chan & Hou (C&H) Medical Laboratories Ltd. (Hong Kong) to exclude those subjects with the possibility of organic disorders and metabolic diseases. The information of demographics and characteristics of all subjects is described in Table 2. Serum samples were extracted from fasting blood and immediately stored at −20°C in the C&H lab. Serum samples were daily delivered on dry ice to the laboratory in School of Chinese Medicine by members of our research group.

**Table 2:** Demographics and clinical characteristics of healthy human populations.

**Characteristics**	**Values (mean ±SEM)**	**Normal range**
Gender	F/M (68/22)	
Age (years)	39.8 ± 1.3	
BMI (kg/m^2^)	21.3 ± 0.3	
ALP (U/L)	63.7 ± 1.7	38–126
ALT (U/L)	18.1 ± 1.3	5–40
AST (U/L)	24.4 ± 0.6	5–37
Total cholesterol (mmol/L)	4.9 ± 0.1	2.84–5.68
Glucose (mmol/L)	4.6 ± 0.1	3.6–6.1
Triglyceride (mmol/L)	1.0 ± 0.1	0.56–1.7
Creatinine (μmol/L)	63.5 ± 1.3	F: 46–92; M: 58–110
Urea (mmol/L)	4.5 ± 0.1	F: 2.5–6.1; M: 3.2–7.1
Total bile acid (μmol/L)	0.8 ± 0.1	0–10

### Data analysis

The data are presented as mean ± SEM. Statistical differences of bile acids and the corresponding gene expression level between individual groups were evaluated using Student's *t*-test. GraphPad Prism 5.0 software (GraphPad Software Inc., San Diego, CA, USA) was used for the calculations. Less than 0.05 of *p-*value was regarded as significant difference. For the correlation analysis, the data was plotted in a scatter chart with linear regression fit and the respective Pearson correlation coefficients were calculated. Analyses were performed using Prism statistical program and significance threshold was set to 0.05.

## Results

### Alteration in serum metabolome of spexin-injected rats are primarily linked to bile acids

**Figure 1:**
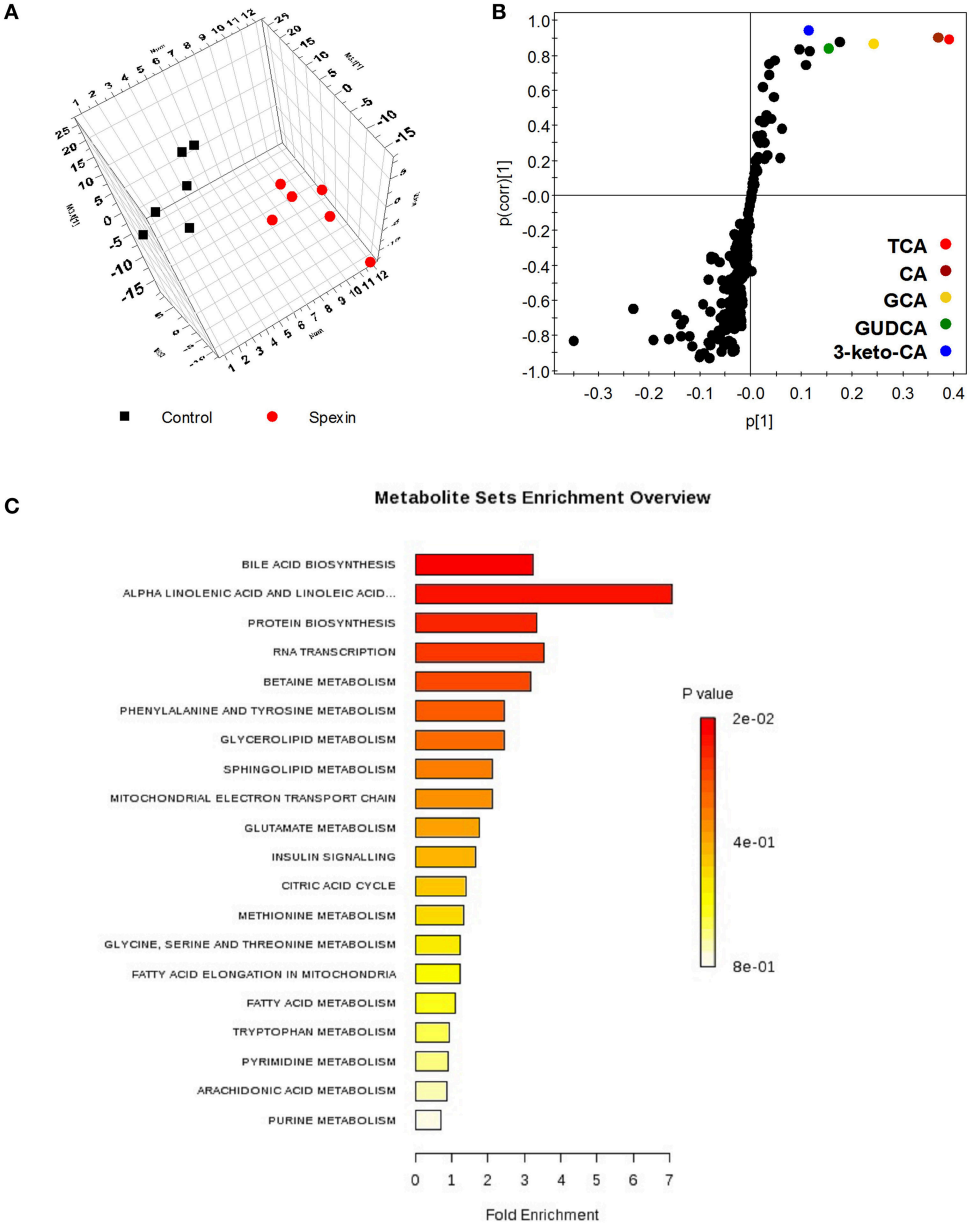
Altered metabolic phenotype induced by spexin in rat serum metabolomic analysis resulting from UPLC/MS with negative ions mode. **(A)** Three dimensional PLS-DA score plot of serum metabolome from control and spexin-injected groups (*n* = 6/group). The parameters of the PLS-DA model are R2X = 0.681, R2Y = 0.756, Q2 = 0.43. **(B)** Scatter chart of metabolic fragments in PLS-DA model reveals the contributing signals for clustering control and treated groups. Each point represents an individual fragment obtained from UPLC/MS acquisition. The labeled metabolites identified as a group of bile acids were significantly decreased by spexin intervention. **(C)** Altered pathway calculated based on metabolic signature in ESI^−^ using MSEA metabolic pathway library conducting on Metaboanalyst (http://www.metaboanalyst.ca/). Bile acid biosynthesis is the top pathway altered by spexin with highest hits and lowest *p*-value (*p* = 0.015).

A total of 640 and 426 features were separately captured under positive and negative ionic modes in global metabolic profiling. Representatively, three-dimensional score chart plotted from PLS-DA model revealed distinct separation of serum metabolomes between the control and spexin-injected groups (Figure 1A). Overall, 40 fragments contributing to cluster metabolic patterns of spexin-treated vs. control rats were identified and are listed in Figure S1 and Table S2. Five ions in the scatter plot significantly decreased in spexin-injected group, were identified as a group of bile acids by MS/MS fragments against endogenous metabolites databases (Figure 1B). Metabolite set enrichment analysis (MSEA) for those changed signatures revealed that bile acid biosynthesis topped in enriched pathways (Figure 1C). Moreover, it was also found that several potential metabolites such as long chain fatty acids and phosphocholines, were obviously increased in serum after spexin injection, closely linked to bile acid metabolism. Taken together, untargeted serum metabolomic profiling demonstrates that bile acid homeostasis was altered by spexin.

### Single injection of spexin altered bile acid metabolism in rats

**Figure 2:**
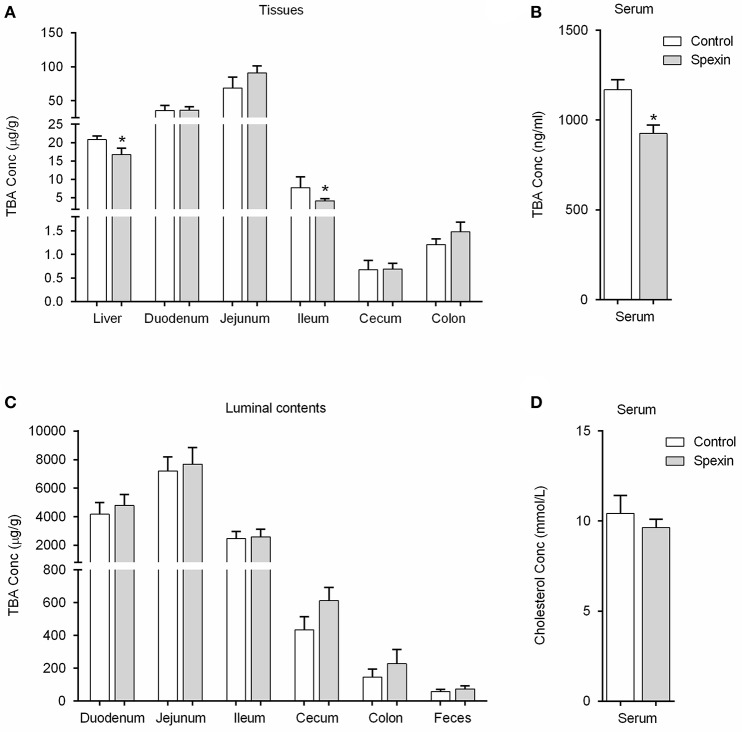
Spexin-induced alteration of bile acid pool in the enterohepatic circulation system. Rats were injected *i.p*. with spexin (300 μg/kg) prepared in saline. One hour later, the rats were sacrificed for tissue sample collection. The rats injected *i.p*. with saline were served as control group. The samples of serum, tissues, luminal contents, and feces were subjected to further bile acid quantification. **(A)** Spexin-induced alteration of the TBA in individual tissues in rat. **(B)** Spexin-induced alteration of the TBA in rat serum. **(C)** Spexin did not alter TBA levels in rat luminal contents and feces. **(D)** Single injection of spexin cannot affect the TC concentration in rat serum. Results are shown as Mean ± SEM (*n* = 8/group). *p < 0.05 vs. control resulting from Student's *t*-test. Effect of spexin on individual bile acids throughout the enterohepatic circulation was also evaluated, see in Figures S1, S2.

To investigate the changes in bile acid composition induced by spexin in rats, the specific bile acids were quantified and compared in different regions of the enterohepatic system. Quantitative analysis on individual metabolites revealed that the levels of the three major BAs including GCA, TCA and TβMCA were significantly reduced in the liver following spexin injection (Figure S2), leading to significant decrease of hepatic TBA (Figure 2A). In ileum, 1-h treatment of spexin could suppress the TβMCA, TCA, TDCA, and THDCA levels (Figure S2) with concurrent decrease of TBA (Figure 2A). However, the TBA level in the ileal content was not affected by spexin (Figure 2A), which may be attributed to the unchanged CA concentration although several individual bile acids were significantly changed (Figure S3). Further, spexin could remarkably reduce the concentrations of TCA, TDCA, TβMCA, GCA, and CA in rat serum (Figure S2). Not surprisingly, the serum TBA level was also significantly suppressed by spexin injection in rats (Figure 2B). In the colon, the CDCA and UDCA were increased by spexin (Figure S2) that may be due to the enhanced bowel motility as previously reported (Lin et al., 2015). However, the TBA was not significantly altered in both colonic content and feces (Figure 2C), indicating that spexin injection did not affect the excretion of bile acids. Besides, no significant difference of the serum TC was noticed between the control group and spexin treatment group (Figure 2D).

### Long-term injection of spexin for 28 days altered total bile acid pool in mice

**Figure 3:**
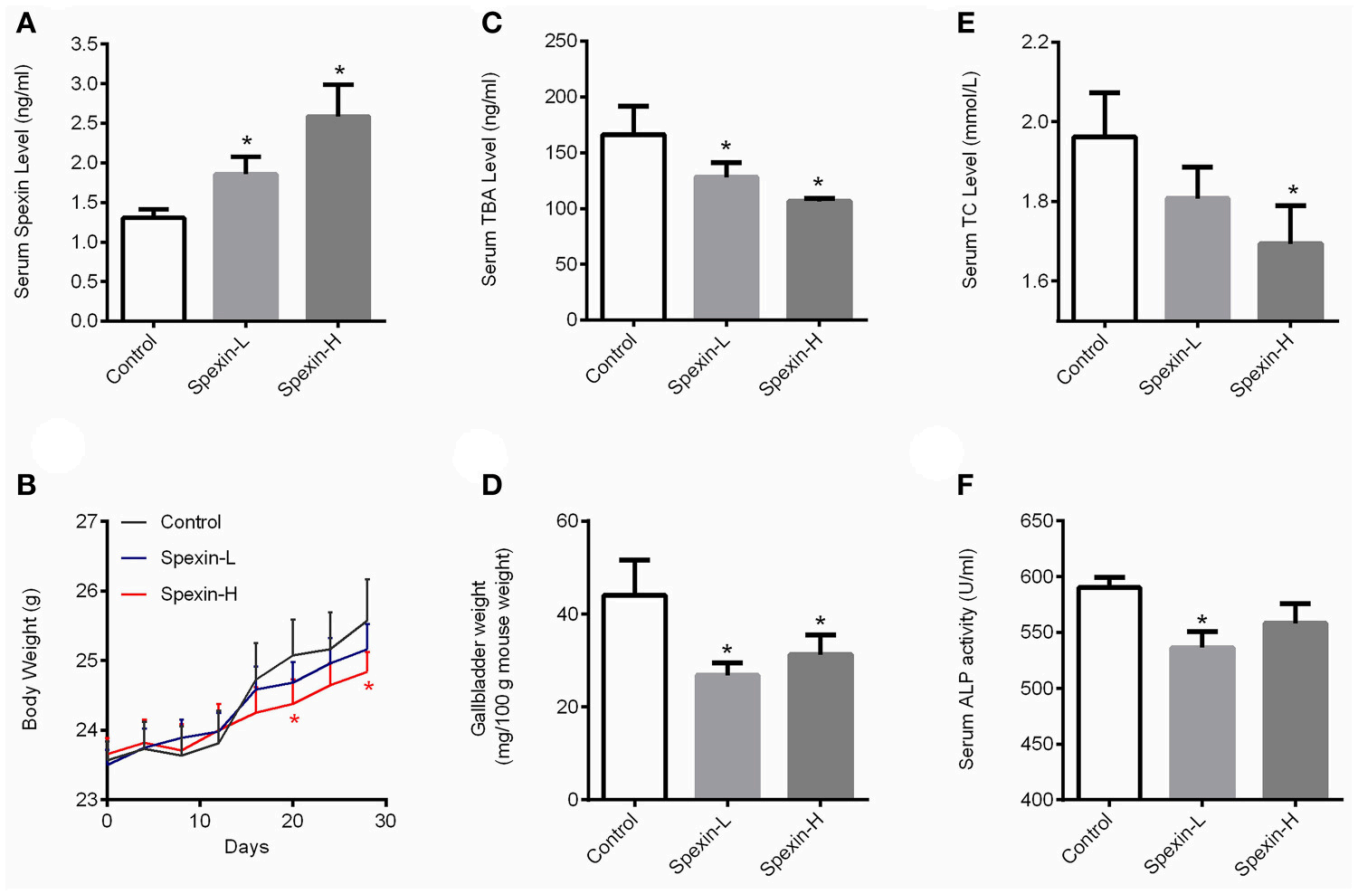
Long-term injection of spexin for 28 days altered total bile acid pool in mice. The mice were *i.p*. injected with spexin (Spexin-L: 12.5 μg/kg and Spexin-H: 25 μg/kg) or saline daily for 28 consecutive days. Body weight was monitored every 4 days. At the end of the experiment, blood, liver, and gallbladder were harvested for further analysis. **(A)** Circulating spexin level in mice with/without spexin injection. **(B)** Spexin (25 μg/kg) inhibited mouse body weight gain. **(C)** Spexin reduced serum TBA concentration in mice. **(D)** Spexin decreased gallbladder size. **(E)** Spexin suppressed serum TC level. **(F)** Spexin inhibited serum ALP acitivity. Results are shown as Mean ± SEM (*n* = 10/group). *p < 0.05 vs. control resulting from Student's *t*-test.

To further investigate the role of spexin in regulating bile acid metabolism, long-term injection with spexin at doses of 12.5 and 25 μg/kg were conducted in mice. As shown in Figure 3A, serum spexin level in untreated mice was recorded as 1.3 ng/mL, while the serum concentration of spexin was increased to 1.8 and 2.6 ng/mL at 1-h post-injection of spexin at 12.5 and 25 μg/kg, respectively. The body gain was significantly slowed down by spexin injection at 25 μg/kg from day 20 (Figure 3B). The results demonstrated that long-term treatment by both low dose and high dose of spexin injection can significantly decrease serum TBA levels (Figure 3C) and gallbladder size (Figure 3D) in mice. Notably, circulating TC and ALP concentrations were also reduced by long-term spexin injection (Figures 3E,F).

### Specific bile acids in liver/gallbladder/ileal contents of spexin-treated and control mice

**Figure 4:**
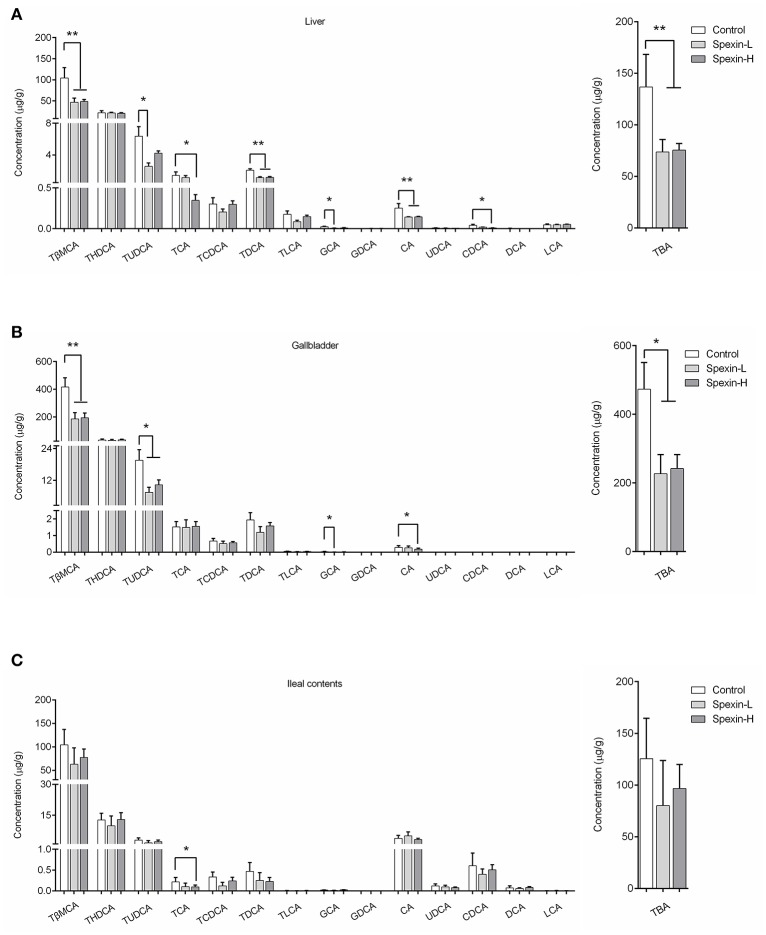
Inhibitory effect of spexin on bile acid metabolites in mice. Specific bile acids were quantified in the liver, gallbladder and ileal contents of male C57BL/6J mice daily *i.p*. injected with 12.5 μg/kg spexin (Spexin-L), 25 μg/kg spexin (Spexin-H) or saline (Control) for 28 consecutive days. Individual bile acid changes were analyzed and TBA concentrations were calculated by sum of all the bile acid measured in the liver **(A)**, gallbladder **(B)**, and ileal contents **(C)**. Results are shown as Mean ± SEM (*n* = 10/group). *p < 0.05 and **p < 0.01 vs. control resulting from Student's *t*-test.

Analysis of individual bile acids revealed that tauro-conjugated bile acids were major bile acid metabolites in the liver, gallbladder, and ileum content (Figure 4). In the ileum content, the percentage of CA and CDCA was dramatically increased due to the deconjugation by gut microbiota. Compared with the control, liver from spexin-injected mice contains reduced levels of TBA reflected by significantly decreased TβMCA, TUDCA, TCA, TDCA, GCA, CA, and CDCA concentration (Figure 4A). Partially resembling that of the liver, the bile acid profile of the gallbladder was changed by spexin in which TBA including TβMCA, TUDCA, GCA, and CA levels were suppressed (Figure 4B). In the ileum, however, only TCA were significantly reduced. Long-term injection of spexin did not alter the TBA level in mice ileum content (Figure 4C), indicating that the effect of spexin on bile acid metabolism mainly occurs in the liver.

### Long-term injection of spexin alters the expression profile of genes involved in bile acid synthesis

**Figure 5:**
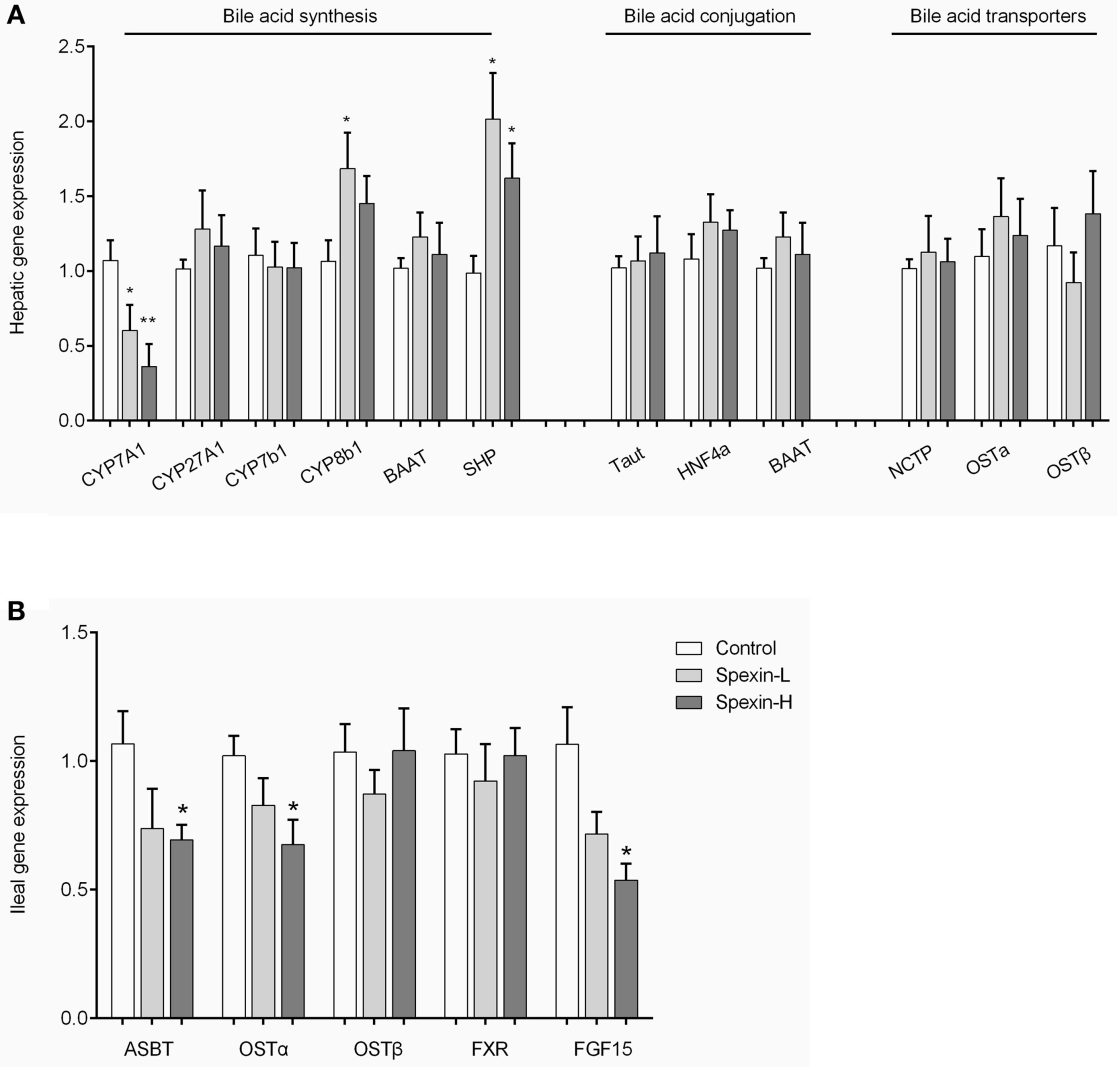
Spexin regulation of genes involved in bile acid synthesis, conjugation, transportation and reabsorption. Gene expression of enzymes involved in bile acid biosynthesis, conjugation, and bile acid transporters in the liver **(A)**, and genes involved in bile acid reabsorption in the ileum **(B)** were quantified by Real-time PCR. Control (*i.p*. injection with saline); Spexin-L (*i.p*. injection with 12.5 μg/kg spexin); Spexin-H (*i.p*. injection with 25 μg/kg spexin). Results are shown as Mean ± SEM (*n* = 10/group). *p < 0.05 and **p < 0.01 vs. control resulting from Student's *t*-test.

To determine whether the altered bile acid profile in spexin-treated mice was associated with the regulation of enzymes in the bile acid synthesis and other related pathways, the mRNA expression of hepatic genes for bile acid synthesis, bile acid conjugation and bile acid transporters, and ileal genes for bile acid reabsorption were quantified by qPCR. Long-term exposure to spexin resulted in dose-dependent reduction in CYP7A1 expression levels in mice liver and significant upregulation of CYP8b1 and SHP (Figure 5A). The expression of sterol regulatory element-binding protein-1c (SREBP-1c) was significantly decreased by high dose of spexin treatment (Figure S4). However, the hepatic expression of the genes for bile acid conjugation and transportation were not significantly affected by spexin injection in mice. In the ileum, gene expressions of ASBT, OSTα, and FGF15 were down-regulated (Figure 5B).

### Spexin suppresses bile acid synthesis via GALR2 and GALR3 receptors in mice

**Figure 6:**
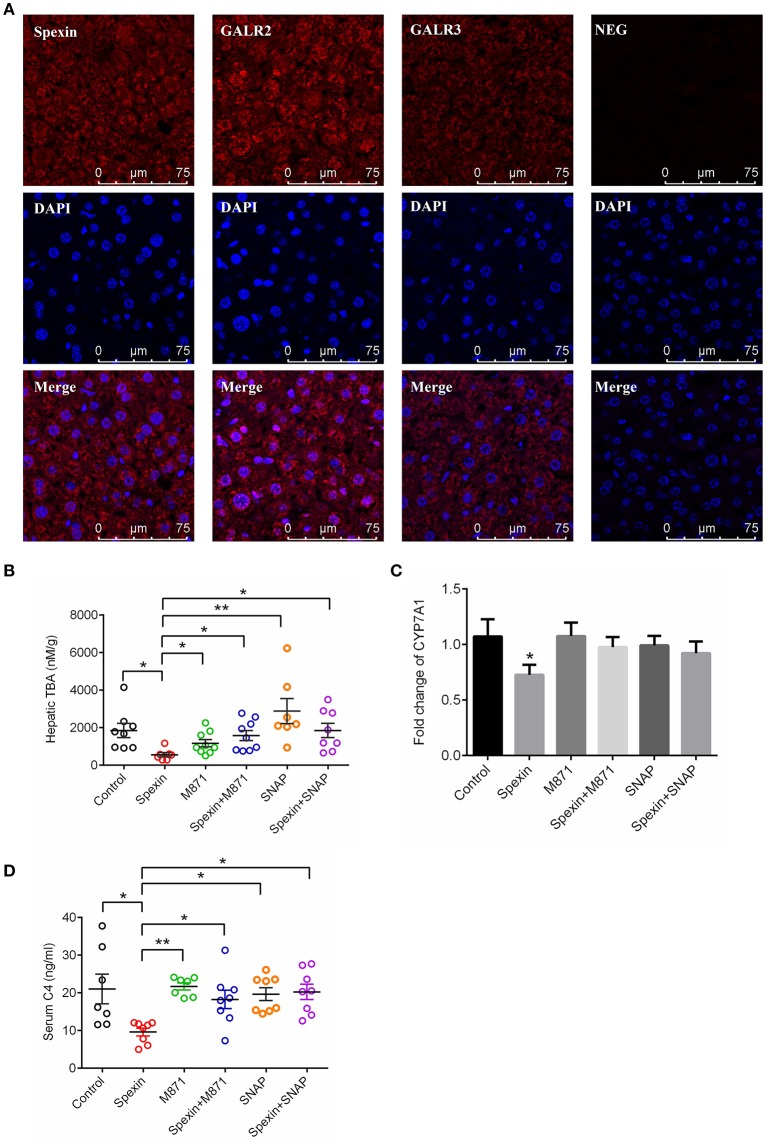
Spexin inhibited bile acid synthesis through GALR2 and GALR3 receptors. **(A)** Expression of spexin, GALR2 and GALR2 in hepatocytes characterized by confocal imaging. To investigate the role of GALR2/3 in spexin-regulated bile acid synthesis, the mice were *i.p*. injected with GALR2/3 antagonists 15 min before spexin treatment. One hour after drug treatment, serum and liver samples from each mouse were collected for further analysis. Control (*i.p*. with saline); Spexin (*i.p*. with spexin 300 μg/kg); M871 (*i.p*. with M871 1000 μg/kg); Spexin+M871 (*i.p*. with M871 15 mins before spexin treatment); SNAP37889 (*i.p*. with SNAP37889 1 mg/kg); Spexin+SNAP37889 (*i.p*. with SNAP37889 15 min before spexin treatment). Hepatic level of TBA **(B)**, hepatic expression of CYP7A1 **(C)** and serum concentration of C4 **(D)** were measured as described. Results are shown as Mean ± SEM (*n* = 10/group). *p < 0.05 and **p < 0.01 between different groups resulting from Student's *t*-test.

Consistent with observations in rats (Porzionato et al., 2010), intense cytoplasmic staining of spexin was found in the hepatocytes of mice, with co-localization with GALR2 and GALR3 receptors (Figure 6A). This suggests spexin may play biofunctions in the liver via the two receptors, GALR2 and GALR3. In the results, we found that both GALR2 antagonist and GALR3 inhibitor could effectively attenuate the inhibitory effect of spexin on hepatic TBA level (Figure 6B) and reverse the suppressed CYP7A1 expression by spexin injection (Figure 6C). The serum concentration of C4 is a marker of CYP7A1 and bile acid synthesis (Axelson et al., 1988). In mice, spexin significantly inhibited serum C4 level which could be effectively abolished by GALR2 and GALR3 blockade (Figure 6D).

### Serum spexin are negatively correlated with total bile acid and major BA metabolites in healthy volunteers

**Figure 7:**
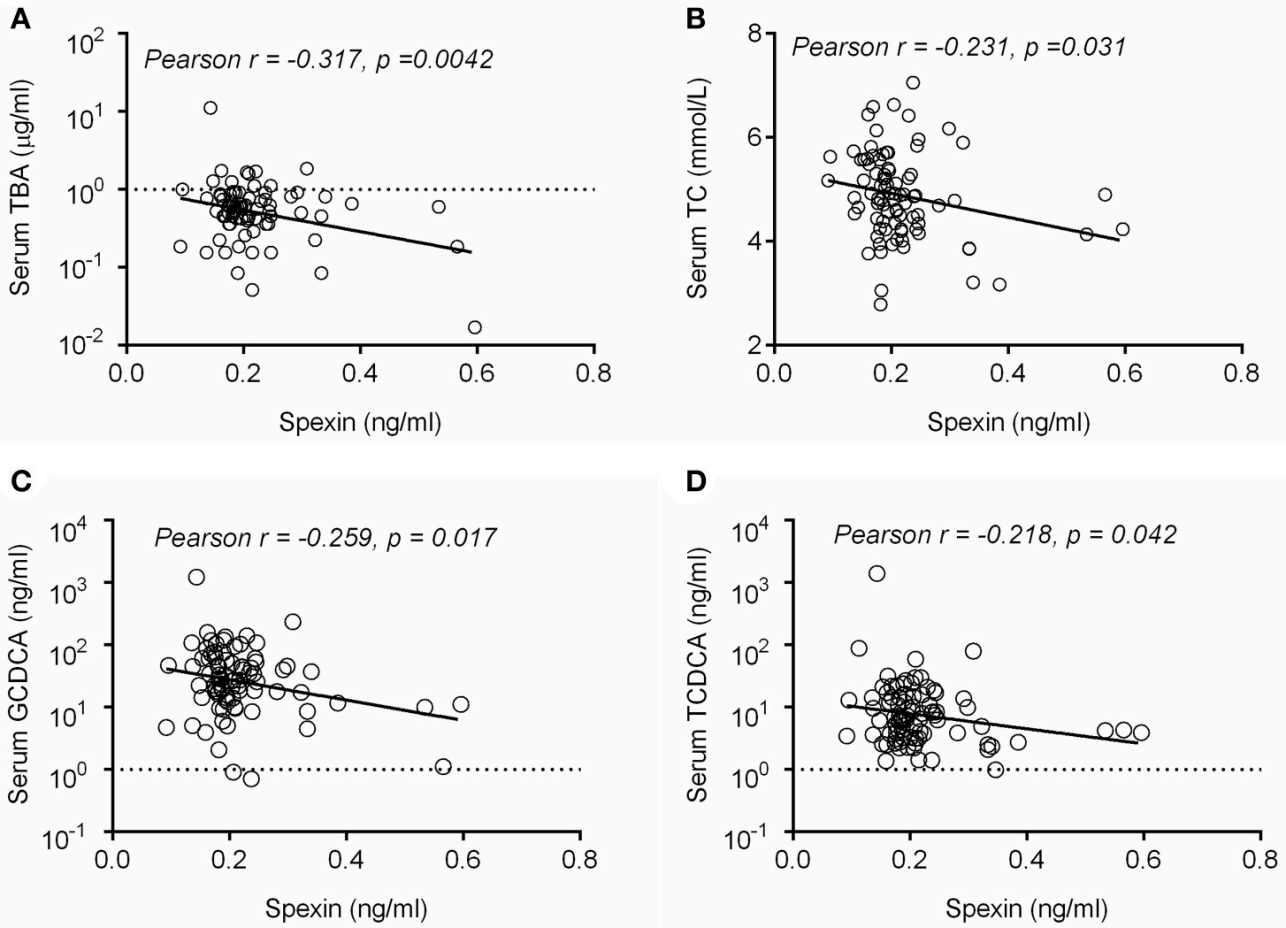
Serum spexin and bile acid levels were negatively correlated in healthy volunteers. Correlation analysis of serum spexin with serum TBA **(A)**, serum TC **(B)**, serum GCDCA **(C)**, and serum TCDCA **(D)** were performed in this study using GraphPad Prism 5.0 software. The data was plotted in a scatter chart with linear regression fit and the respective Pearson correlation coefficients and the *p-*value were calculatí.

To confirm whether spexin is associated with bile acid metabolism in human, we performed a clinical study to determine the relationship between serum spexin and TBA levels in 91 healthy volunteers. In humans, serum spexin was found to be negatively correlated with TBA (*p* = 0.0042, Figure 7A) and TC levels (*p* = 0.0031, Figure 7B). Specifically, significant negative correlations were also observed between spexin and serum levels of GCDCA or TCDCA (Figures 7C,D).

## Discussion

Recent clinical investigations indicate spexin is an important peptide involved in obesity and diabetes (Kumar et al., 2016, 2017; Hodges et al., 2017; Kolodziejskii et al., 2017), consistently supported by the current finding that long-term injection of spexin decreased the gain in body weight, in mice. The spexin receptor, GALR3, was shown to be involved in the modulation of cholesterol and triglyceride levels in mice (Brunner et al., 2014). Furthermore, spexin was found to effectively reduce hepatic total lipids (Ge et al., 2016), suggesting its important role in lipid metabolism in mammals. In this study, spexin injection significantly reduced serum cholesterol level in mice and rats. By global metabolic profile analysis of rat serum, we found lipid metabolism and the related metabolic pathway were remarkably altered by spexin. Among the potential metabolic markers induced by spexin in rat serum, a group of bile acids were significantly decreased, indicating the intimacy of spexin with bile acid signaling.

To fully understand the effects of spexin on bile acid circulating pool, we systematically examined the bile acid profiles in serum, liver, gastrointestinal tract tissues and contents, and feces in rats. By spexin injection, the bile acid pool in enterohepatic circulation was altered. Particularly, the hepatic and serum bile acids exhibited much higher sensitivity against spexin injection. In contrast, the TBA was not significantly affected in the intestine, colon, and feces, although some individual bile acids were changed. These results indicate that the liver may be the prime organ where spexin regulates bile acid metabolism. One limitation is that changes of bile acids in the rat serum were measured at single time point. We cannot describe the whole process of bile acid alterations after spexin treatment. The conclusion could be more informative by measuring the changes of bile acid metabolites at multiple time points after acute spexin treatment.

In the liver, cholesterol is catalyzed to primary bile acid CA and CDCA mainly by the enzyme CYP7A1, which is called classic or neutral pathway. CA and CDCA are further conjugated with either taurine or glycine catalyzed by BAAT and BACS to form tauro-conjugated or glycine-conjugated bile acids (Lefebvre et al., 2009). The transporters MRP2, MRP4, and OSTα/β provide excretion routes for bile acids into the systemic circulation (Thomas et al., 2008; Chiang, 2009). In mice, chronic treatment of spexin significantly suppressed CYP7A1 gene expression in the liver. Meanwhile, bile acid conjugation and transporters were not involved in the inhibitory effect of spexin on bile acid pool. It is known that CYP7A1 expression can be suppressed by several intrinsic hepatic factors including SHP (Chiang, 2009) and SREBP-1c, a non-DNA-binding transcriptional inhibitor of CYP7A1 gene expression (Ponugoti et al., 2007). Our results showed that spexin can significantly increase the expression of SHP while decrease the expression of SREBP-1c in mouse liver, indicating that spexin may suppress CYP7A1 expression by upregulating hepatic SHP level instead of SREBP-1c. Alternatively, the inhibitory effect of spexin on SREBP-1c may be involved in the weight loss by downregulating the expression of genes for specific LCFA transporters (Ge et al., 2016). Furthermore, the TBA and C4 levels as well as hepatic CYP7A1 mRNA expression were significantly inhibited by 1-h spexin treatment. GALR2 and GALR3 blockades could effectively abolish the suppression of spexin on hepatic TBA and CYP7A1 expression levels, in coordination with changes in serum C4 level. These results suggest that the spexin-induced bile acid profile alteration may be caused by the suppressed bile acid synthesis.

Bile acids are deconjugated, dehydrogenated, and dehydroxylated in the ileum and colon (Ridlon et al., 2006), causing a dramatic decrease in tauro-conjugated bile acids and increase in primary bile acids (CA and CDCA) proportions, which is replicated in current results in mice and rat. Long-term injection with spexin did not significantly alter bile acid pool in mice ileum, ruling out intestine as the prime organ for spexin regulation on bile acid metabolism. In ileum, bile acids are reabsorbed by ASBT and effluxed by OSTα/β into the portal vein (Thomas et al., 2008). Simultaneously, ileum bile acids can activate FXR to modulate circulating FGF15 (FGF19 in humans) levels, thereby regulating bile acid biosynthesis through feedback mechanism (Li et al., 2014; Li and Chiang, 2015). In our results, the inhibitory effects of spexin on ASBT, OSTα, and FGF15 gene expression were observed. Physiologically, the decreased FGF15 production will attenuate the negative regulation on CYP7A1 expression by classic feedback loop (Chiang, 2009). In this case, altered ileal FGF15 gene expression may not contribute to the inhibitory effect of spexin on bile acid pool. The repression of spexin on bile acid metabolism could be due to the down-regulation of bile acid synthesis in the liver. Alternatively, the decreased ileal concentration of TCA, an endogenous FXR activator (Cyphert et al., 2012), may account for the reduced expression levels of ASBT, OSTα, and FGF15.

In rats, phospholipid metabolites obviously increased in serum after acute spexin injection, linking with the previous report that spexin could reduce the uptake of long chain fatty acids by adipocytes in rats (Walewski et al., 2014). In the mice, we found that chronic spexin treatment can significantly decrease serum TC level, which may also associate with the anti-obesity function of spexin. However, the serum TC level was not affected by acute spexin treatment in rats. These results suggest that there should be some differences on the changes of metabolomic profile between acute and chronic treatment. In the case of bile acid metabolites, hepatic levels of GCA, TCA, and TβMCA were suppressed by acute spexin treatment, while much more bile acids (TβMCA, TUDCA, TCA, TDCA, GCA, CA, CDCA) were inhibited by chronic spexin treatment. Further metabolomic assay in the serum and tissue samples after chronic treatment may help to fully understand the effect of spexin on the intrinsic metabolic pathways.

Our clinical study demonstrated that spexin is negatively correlated with serum TBA and TC, reinforcing spexin's existing biofunction profile in regulating bile acid metabolism. In humans, glyco-conjugated bile acids are predominant in serum especially GCDCA (Wang et al., 2016), which is also found negatively correlated with spexin in this study. These results suggest that spexin may be an important intrinsic suppressor for bile acid synthesis. Recent studies demonstrated that bile acids can activate G protein-coupled bile acid receptor 1 (TGR5) to regulate energy homeostasis and reduce body weight (Watanabe et al., 2006; Chen et al., 2017), suggesting that both spexin and bile acids serve as negative regulators for the body weight gain, for which the effect of spexin on mice body weight loss should be independent of bile acid signaling. Nevertheless, spexin may induce significant body weight loss by reducing caloric intake, increasing energy expenditure, altering the respiratory exchange ratio that increases burning of fat, and inhibiting LCFA uptake into both human and mouse adipocytes and mouse hepatocytes (Ge et al., 2016). Dysregulation of the enterohepatic bile acid circulation has been suggested to contribute to the pathogenesis of non-alcoholic fatty liver disease (NAFLD), obesity, diabetes, dyslipidemia, arteriosclerosis, and gut inflammation (Thomas et al., 2008; Lefebvre et al., 2009; Banerjee et al., 2016). Therapeutic targeting of bile acid pathways may provide new perspectives for the treatment of these disorders. Thus, further studies may help understand the actions of spexin on bile acid pathway, and the pathogenesis of the diseases associated with the dysbiosis of bile acid metabolism.

**Figure 8:**
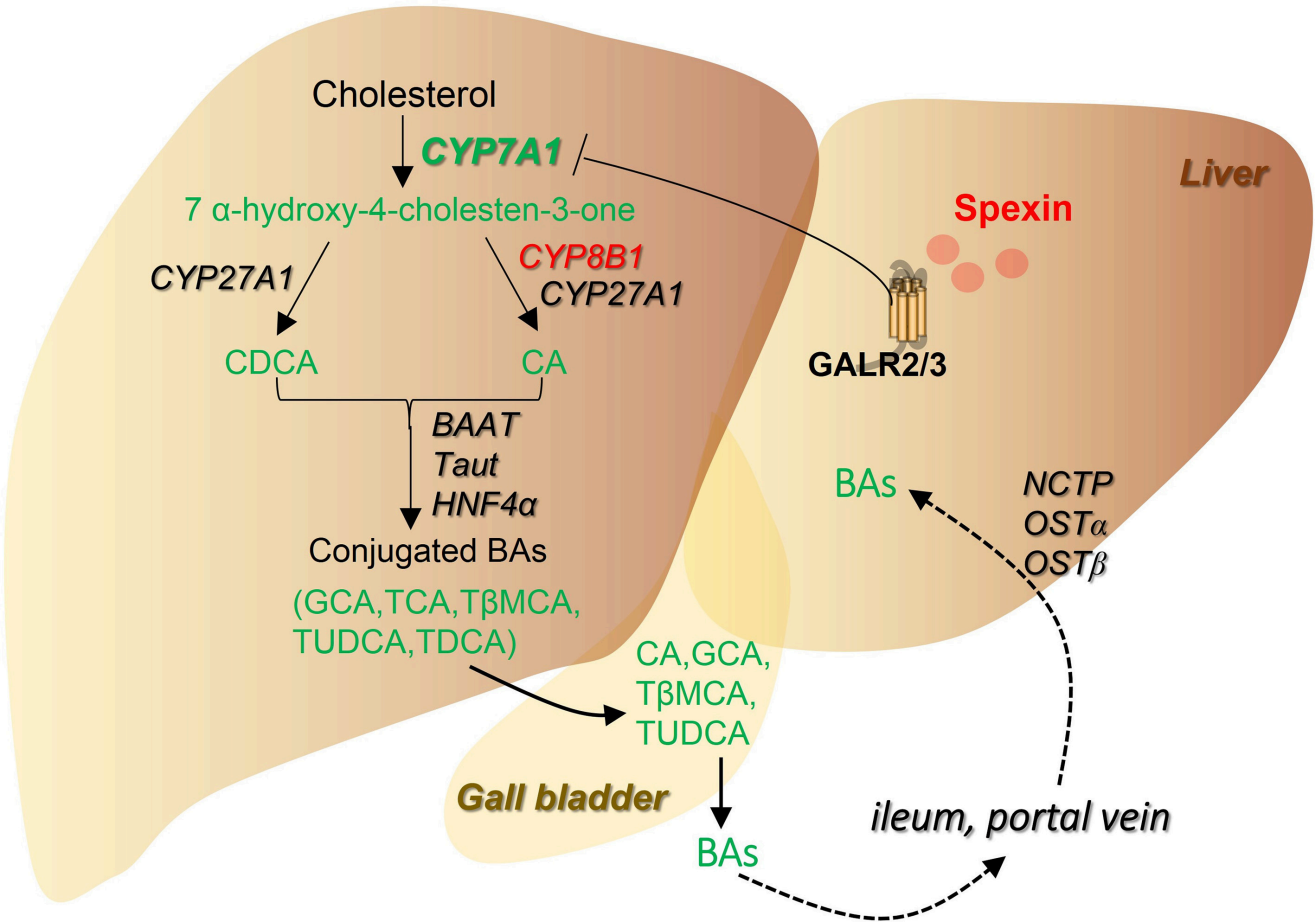
Summarized scheme for the regulation of spexin on bile acid pathway. Changes of the metabolites and genes were highlighted (Red, increased; Green, decreased). Spexin downregulates bile acid synthesis by suppressing CYP7A1 expression, resulting in a decrease of total bile acid pool in the enterohepatic circulation.

In summary, global metabolic profile revealed multiple metabolic pathways altered by spexin which can provide vital clues for further biofunction studies. More importantly, spexin was found to play crucial roles in bile acid metabolism by inhibiting bile acid synthesis via GALR2/3 receptors (summarized in Figure 8), in coordination with the clinical finding that spexin was negatively correlated with serum bile acid levels of healthy populations.

## Author contributions

CL and LZ: performed the majority of experiments, data acquisitions, analyzed data, and wrote the manuscript; TH: assisted with *in vitro* experiments and helped analyze results; LL: contributed to confocal imaging; LLZ: contributed to clinical sample collection; JL: contributed to Real-time PCR analysis; MK: contributed to the writing and revising of the manuscript; ZC: contribute to the bile acid standards and metabolic analysis; BF: contributes to metabolites detection and experimental tools; AW: contribute to the animal experiments and provide guidance to the study; ZB and CL: designed the experiment, supervised the study, and contributed to finalize the manuscript. All authors reviewed the manuscript.

### Conflict of interest statement

The authors declare that the research was conducted in the absence of any commercial or financial relationships that could be construed as a potential conflict of interest.

## Abbreviations

ASBT: apical sodium dependent bile acid transporter
BAAT: bile acid CoA: amino acid N-acyltransferase
BACS: bile acid CoA synthetase
BSEP: bile salt export pump
C4: 7 α-hydroxy-4-cholesten-3-one
CA: cholic acid
CDCA: chenodeoxycholate
CYP7A1: cholesterol 7α-hydroxylase 1
CYP8B1: sterol 12α-hydroxylase
CYP27A1: sterol 27-hydroxylase
DCA: deoxycholate
FGF15: fibroblast growth factor 15
FGFR4: fibroblast growth factor receptor 4
FXR: farnesoid X receptor
GCA: glycocholate
GCDCA: glycochenodeoxycholic acid
GDCA: glycodeoxycholate
HNF4α: hepatocyte nuclear factor 4 alpha
IS: internal standard
MS: mass spectrometry
NIR: near-infrared
NTCP: sodium-taurocholate cotransporting polypeptide
OSTα/β: organic solute transporter α/β
PCA: principal component analysis
SHP: small heterodimer partner
Tβ-MCA: tauroβ-muricholate
TCA: taurocholate
TCDCA: taurochenodeoxycholate
TDCA: taurodeoxycholate
TGR5: G protein–coupled bile acid receptor 1
THDCA: taurohyodeoxycholate
TLCA: taurolithocholate
TUDCA: tauroursodeoxycholate
TBA: total bile acid
UPLC: ultraperformance liquid chromatography.
