# Supplementary material for: Spexin Acts as Novel Regulator for Bile Acid Synthesis
Source: Front Physiol. 2018 Apr 10;9:378. doi: 10.3389/fphys.2018.00378 (PMC5902714; doi:10.3389/fphys.2018.00378)
Supplement: Supplementary file 1 [file DataSheet1.DOCX]

Table S1. MRM transitions and MS parameters for the analytes and IS

| Bile acid | Parent (m/z) | Daughter (m/z) | Dwell time (s) | Frag (V) | Collision (V) | Polarity |
| --- | --- | --- | --- | --- | --- | --- |
| TβMCA | 514 | 80 | 25 | 250 | 87 | Negative |
| TCA | 514 | 80 | 25 | 250 | 87 | Negative |
| TDCA | 498 | 80 | 25 | 192 | 80 | Negative |
| TUDCA | 498 | 80 | 25 | 192 | 80 | Negative |
| THDCA | 499 | 80 | 25 | 192 | 80 | Negative |
| TCDCA | 498 | 80 | 25 | 192 | 80 | Negative |
| TLCA | 482 | 80 | 25 | 220 | 80 | Negative |
| GCA | 464 | 74 | 25 | 250 | 39 | Negative |
| GDCA | 448 | 74 | 25 | 250 | 35 | Negative |
| GCDCA | 448 | 74 | 25 | 250 | 35 | Negative |
| CA | 407 | 407 | 25 | 250 | 10 | Negative |
| UDCA | 391 | 391 | 25 | 250 | 10 | Negative |
| CDCA | 391 | 391 | 25 | 250 | 10 | Negative |
| DCA | 391 | 391 | 25 | 250 | 10 | Negative |
| LCA | 375 | 375 | 25 | 250 | 10 | Negative |
| DCA-d4 | 395 | 349 | 25 | 250 | 35 | Negative |
| C4 | 401 | 177 | 200 | 200 | 23 | Positive |

Table S2. Significant altered features resulted from serum metabolic profile between control and spexin-injected groups

| Pathway | Class ^a^ | Metabolite | FC value ^b^ | VIP | p value | q value |
| --- | --- | --- | --- | --- | --- | --- |
| Amino acid metabolism | Amino acid | L-Tryptophan | -0.31 | 1.37 | 0.029 | 0.104 |
|  |  | L-Tyrosine | -0.24 | 1.04 | 0.048 | 0.123 |
|  |  | L-Phenylalanine | -0.38 | 1.20 | 0.069 | 0.146 |
|  | Carboxylic acid | N-Acetyl-L-glutamic acid | -0.23 | 1.97 | 0.026 | 0.099 |
|  |  | Betaine | -0.39 | 1.45 | 0.050 | 0.126 |
|  | Cinnamic acid | trans-Cinnamic acid | -0.26 | 1.42 | 0.037 | 0.111 |
|  | Indole | Indoleacrylic acid | -0.29 | 1.77 | 0.050 | 0.126 |
| Bile acid metabolism | Bile acid | Tauroursodeoxycholic acid | -4.61 | 1.30 | 0.018 | 0.089 |
|  |  | Glycoursodeoxycholic acid | -1.36 | 1.08 | 0.019 | 0.091 |
|  |  | Taurocholic acid | -2.84 | 1.05 | 0.021 | 0.094 |
|  |  | Glycocholic Acid | -1.44 | 1.17 | 0.030 | 0.105 |
|  |  | 3-Oxocholic acid | -1.48 | 1.30 | 0.036 | 0.110 |
|  |  | Cholic acid | -1.02 | 1.29 | 0.041 | 0.114 |
| Energy metabolism | Carboxylic acid | Succinic acid | -0.74 | 2.12 | 0.014 | 0.086 |
| Lipid metabolism | Branched fatty acid | 3-Methylglutaric acid | -0.36 | 1.88 | 0.035 | 0.108 |
|  | Endocannabinoid | Arachidonoyl dopamine | -1.23 | 1.27 | 0.045 | 0.119 |
|  | Long chain fatty acid | Arachidonic Acid | 0.52 | 1.39 | 0.023 | 0.096 |
|  |  | Linoleic acid | 0.52 | 1.32 | 0.032 | 0.106 |
|  |  | Palmitic acid | 0.39 | 1.08 | 0.046 | 0.120 |
|  |  | Oleic Acid | 0.39 | 1.05 | 0.047 | 0.122 |
|  |  | 2-hydroxy stearic acid | 0.37 | 1.23 | 0.047 | 0.122 |
|  |  | 2-hydroxy hexadecanoic acid | 0.39 | 1.23 | 0.048 | 0.123 |

| Pathway | Class ^a^ | Metabolite | FC value ^b^ | VIP | p value | q value |
| --- | --- | --- | --- | --- | --- | --- |
| Lipid metabolism | Prostaglandin | PGF1α | -1.42 | 1.55 | 0.036 | 0.110 |
|  | Phosphocholine | Phosphocholine | 0.41 | 1.26 | 0.048 | 0.123 |
|  |  | Phosphorylcholine | -0.29 | 1.76 | 0.042 | 0.115 |
|  |  | PC(13:0) | 0.30 | 1.31 | 0.038 | 0.112 |
|  |  | PC(15:0) | 0.43 | 1.35 | 0.032 | 0.106 |
|  |  | PC(17:0) | 0.52 | 1.25 | 0.044 | 0.117 |
|  |  | PC(17:1) | 0.41 | 1.43 | 0.018 | 0.089 |
|  |  | LysoPC(15:0) | 0.32 | 1.27 | 0.052 | 0.129 |
|  |  | LysoPC(20:0) | 0.48 | 1.46 | 0.053 | 0.130 |
|  |  | LysoPC(16:0) | 0.24 | 1.09 | 0.059 | 0.135 |
|  | Phosphoethanolamine | LysoPE(20:4) | 0.27 | 1.59 | 0.029 | 0.104 |
|  |  | LysoPE(22:6) | 0.35 | 1.51 | 0.041 | 0.114 |
|  | Sphingolipid | Sphingosine-1-phosphate | 0.30 | 1.39 | 0.023 | 0.095 |
|  | Steroid | Cholesterol sulfate | -1.96 | 1.39 | 0.048 | 0.123 |
| Nucleotide metabolism | Azolidine | Allantoin | -0.53 | 1.64 | 0.030 | 0.105 |
|  | Purine | Adenine | -0.37 | 1.89 | 0.034 | 0.107 |
|  | Pyrimidine | Cytidine monophosphate | 0.36 | 2.14 | 0.013 | 0.085 |
|  |  | Cytosine | -0.29 | 2.02 | 0.021 | 0.094 |

1. The compounds are classified based on HMDB database (<http://www.hmdb.ca/>);
2. Fold change equals the fold difference in metabolite intensity observed between spexin-treated and control sample, with positive value (> 0) indicating up-regulated change and negative value (< 0) meaning down-regulated change in treatment group comparing to control.
