# Supplementary material for: Spexin Acts as Novel Regulator for Bile Acid Synthesis
Source: Front Physiol. 2018 Apr 10;9:378. doi: 10.3389/fphys.2018.00378 (PMC5902714; doi:10.3389/fphys.2018.00378)
Supplement: Supplementary file 2 [file DataSheet2.PDF]

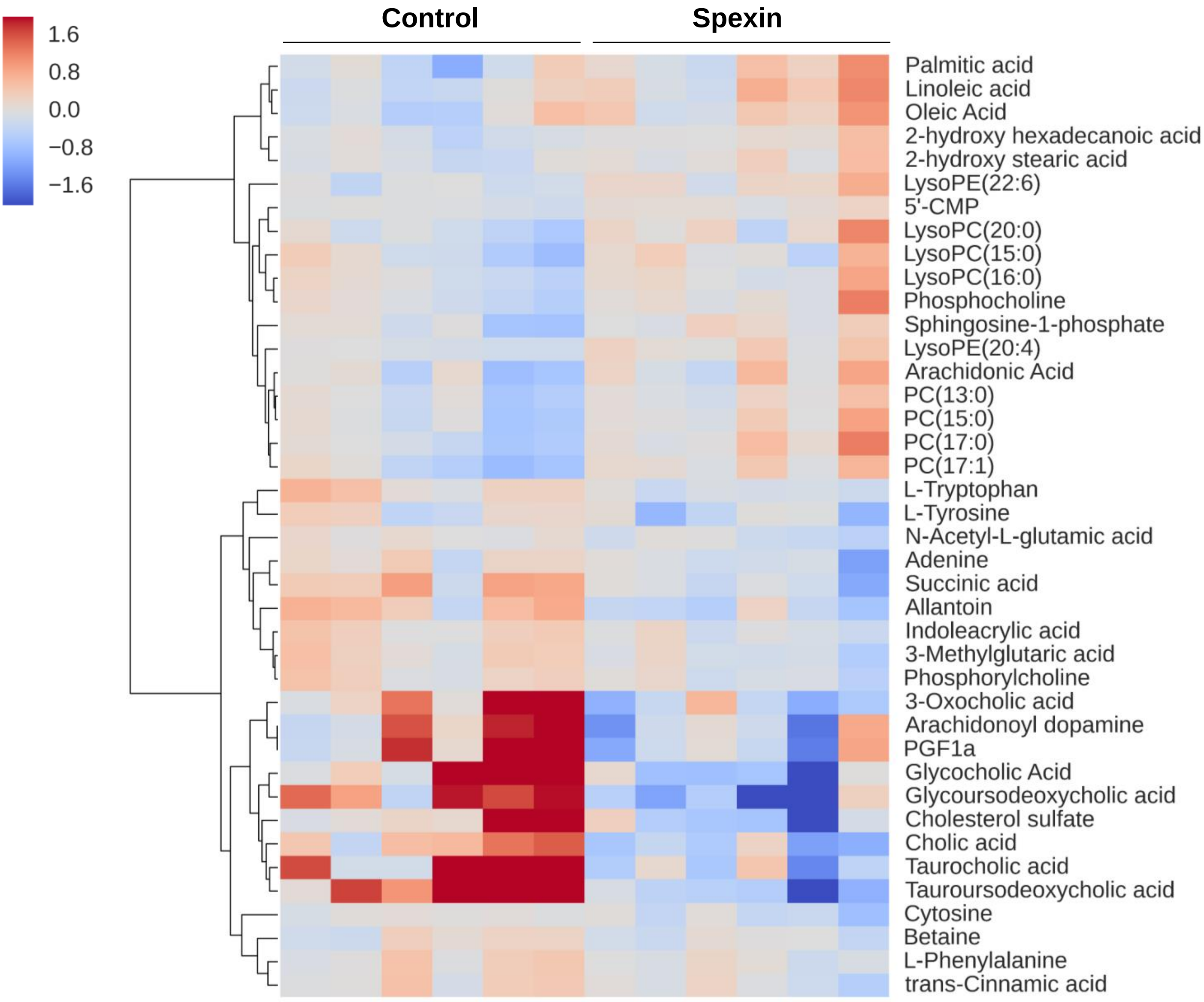

Supplementary Figure 1. Heat map of changes in 40 metabolites levels in the serum of rats. Samples were collected at 1 hour after *i.p.* injection of spexin (300 μg/kg). The color of each section is proportional to the significance of change of metabolites (red, upregulated; blue, downregulated). Rows: samples; Columns: metabolites.

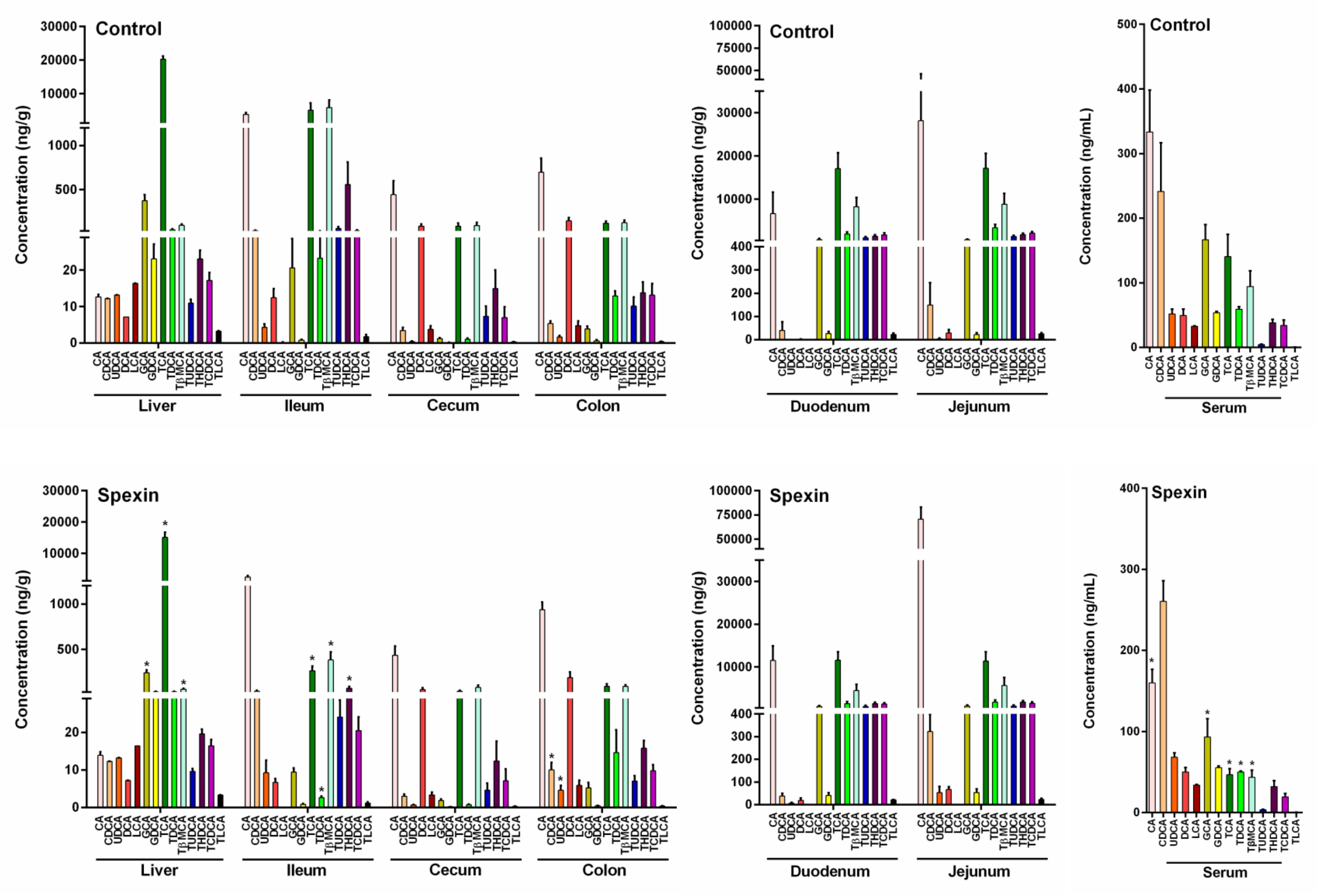

Supplementary Figure 2. Spexin-induced alteration of the specific bile acids in individual tissues/organs and serum in enterohepatic circulation system. Upper: concentrations of the specific bile acids in the rats of control group. Bottom: concentrations of the specific bile acids in the rats of spexin-injection (300 μg/kg). Results are shown as Mean ± SEM (n = 8/group). * $p < 0.05$ versus control resulting from Student's t-test.

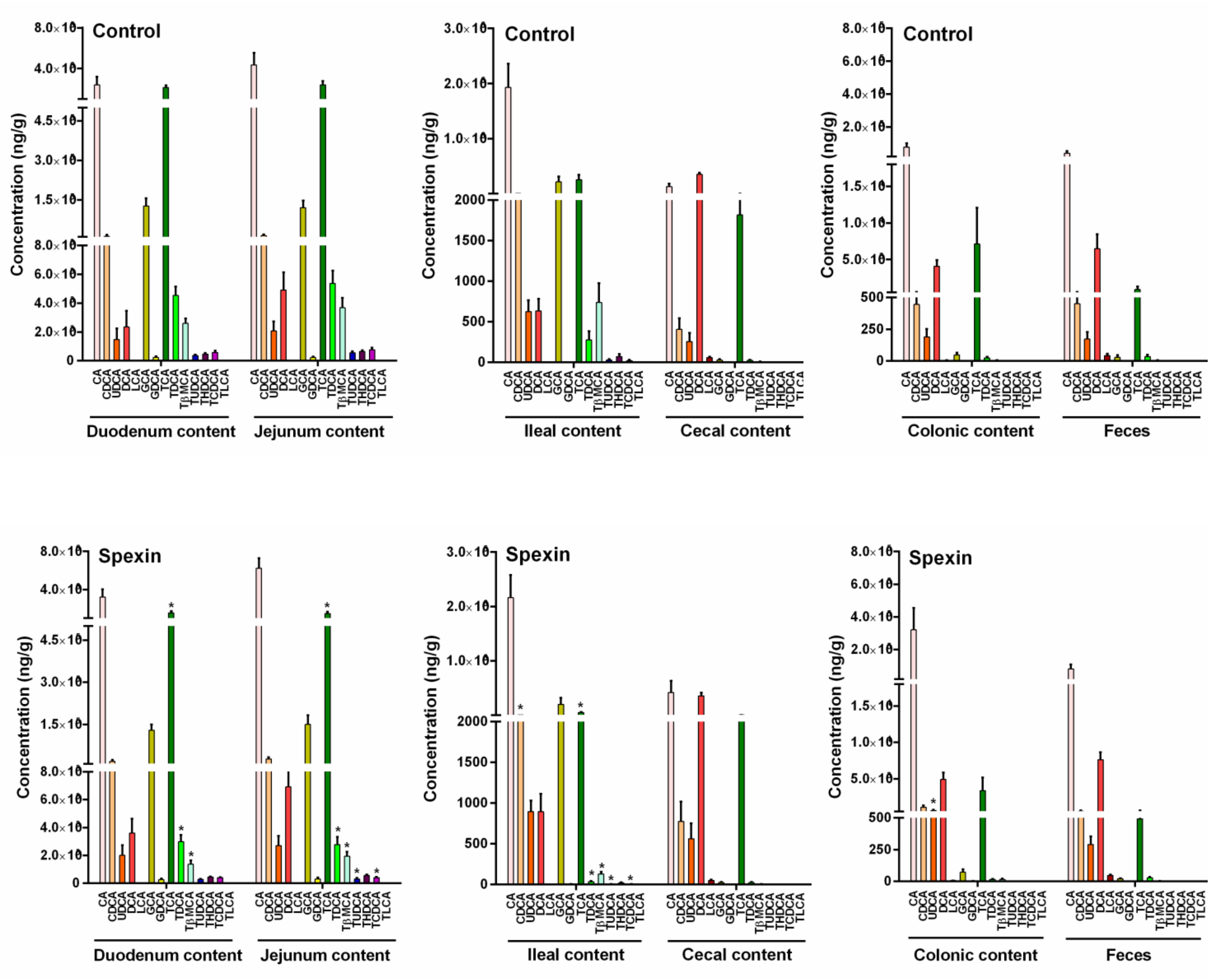

Supplementary Figure 3. Spexin-induced alteration of the specific bile acids in rat luminal contents. Upper: concentrations of the specific bile acids in the rats of control group. Bottom: concentrations of the specific bile acids in the rats of spexin-injection (300 μg/kg). Results are shown as Mean ± SEM (n = 8/group). * $p < 0.05$ versus control resulting from Student's t-test.

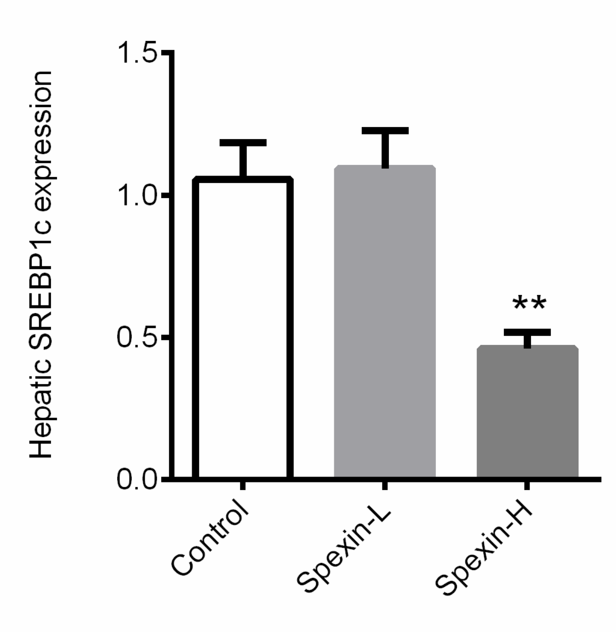

Supplementary Figure 4. Spexin regulation of SREBP1c gene expression in the liver of mice. Control (i.p. injection with saline); Spexin-L (*i.p.* injection with 12.5 μg/kg spexin); Spexin-H (*i.p.* injection with 25 μg/kg spexin). The liver samples were isolated from the mice *i.p.* injected with spexin for 28 days and subjected for total RNA isolation and Real-time PCR quantification. Results are shown as Mean ± SEM (n = 10/group). * * $p < 0.01$ versus control resulting from Student's t-test.
